# Striking life events associated with primary breast cancer susceptibility in women: a meta-analysis study

**DOI:** 10.1186/1756-9966-32-53

**Published:** 2013-08-13

**Authors:** Yan Lin, Changjun Wang, Ying Zhong, Xin Huang, Li Peng, Guangliang Shan, Ke Wang, Qiang Sun

**Affiliations:** 1 Department of Breast Disease, Peking Union Medical College Hospital, Peking Union Medical College, 1 Shuaifuyuan, Wangfujing, Beijing 100730, China; 2 Department of Epidemiology, Insititue of Basic Medical Sciences, Peking Union Medical College, Beijing 100730, China

**Keywords:** Breast cancer, Striking life events, Severe life events, Risk

## Abstract

**Purpose:**

The association between striking life events, an important stress and acute anxiety disorder, and the occurrence of primary breast cancer is unclear. The current meta-analysis was designed to assess the relationship between striking life events and primary breast cancer incidence in women.

**Methods:**

Systematic computerized searching of the PubMed, ScienceDirect, Embase, and BMJ databases with the combinations of controlled descriptors from Mesh, including *breast cancer, breast tumor, cancer of breast, mammary carcinoma, life events, life change events, case–control studies, case-base studies*, *cohort study, and cohort analysis* and identified a total of 307 papers published from January 1995 to April 2012. Following evaluation of methodological quality with the Downs & Black criteria, seven case–control or cohort studies were selected and the association between striking life events and primary breast cancer incidence in women was measured using random effect or fixed-effect odds ratios combined with 95% confidence interval.

**Results:**

The seven studies included in the final meta-analysis included 99,807 women. A meta-analysis showed that the pooled OR for striking life events and breast cancer was 1.51 (95% CI 1.15 - 1.97, *P* = 0.003), indicating that women with striking life events were at 1.5-fold greater risk of developing breast cancer. The pooled OR for severe striking life events and breast cancer was 2.07 (95% CI 1.06 - 4.03), indicating that women with severe striking life events were at 2-fold greater risk of developing breast cancer.

**Conclusions:**

The current meta-analysis showed significant evidence for a positive association between striking life events and primary breast cancer incidence in women.

## Introduction

Primary breast cancer is one of the main public health problems worldwide. Over 1.3 million women are diagnosed annually with primary breast cancer and approximately 458,000 will die from the disease [1]. Various risk factors for primary breast cancer have been identified [2,3], with environmental and life style factors being important [3,4]. In contrast, the association between stress and breast cancer occurrence is unclear, with several cohort studies demonstrating a positive association [5-8] but other studies showing no association [9,10].

An important stress disorder, called striking life events, has been classified as an acute anxiety disorder. This disorder is characterized by aversive anguishing experiences and physiological responses that develop after exposure to stressful life events, including change in marital status, such as separation, divorce, or widowhood; death of a spouse, child, or close relative; a friend’s illness; personal health problems; and change in financial status. This disorder has short-term features, distinguishing it from chronic or delayed-onset stress disorder [11-13]. A prospective cohort study found that chronic stressful life events in women were associated with an increased incidence of breast cancer, with the latter due to chronic stress-induced inhibition of estrogen synthesis, thus explaining the increased incidence of breast cancer in women exposed to long-term high degrees of stress [8]. By contrast, no case–control or cohort study performed to date has assessed the correlation between short-term exposure to stressful life events and the incidence of primary breast cancer.

Conflicting results regarding the association between stressful life events and breast cancer may be due to differences in subject population, number of subjects, study type, and sample type. These findings suggested the need for a meta-analysis examining the relationship between striking life events and primary breast cancer incidence in women.

## Methods

### Purpose

A systematic review and meta-analysis of primary cohort and case–control studies addressed whether women exposed to stressful life events are at increased risk of developing breast cancer. Hence, the objective was to evaluate the association between striking life events and primary breast cancer in women. The use of human materials was approved by the Peking Union Medical College Hospital Medical Ethics Committee (No.S-406).

### Study identification and selection

Eligible studies were identified by systematic computerized searching of the PubMed, Science Direct, Embase, and BMJ databases for relevant reports published from January 1995 to April 2012. The database search strategy used combinations of controlled descriptors from Mesh, including *breast cancer, breast tumor, cancer of breast, mammary carcinoma, life events, life change events, case–control studies, case-base studies*, *cohort study, and cohort analysis*. The reference lists of the retrieved articles were also reviewed to identify additional articles missed by this search.

The following inclusion criteria were adopted for the studies: (a) type of study – prospective and historical cohort and case–control studies; (b) type of sample – women aged 20 years or older at first occurrence of breast cancer in cohort studies and women with the appearance of first breast cancer in case–control studies; (c) mean follow-up time – a minimum of ten years in cohort studies, with no limit between exposure and diagnosis in case–control studies; (d) type of variables – studies in which the stress variable was measured quantitatively, using a numerical scale, questionnaire, or checklist, with stress assessed by measuring the frequency of exposure and intensity of the event; (e) statistical type and analysis – studies that calculated relative risk (RR) for the first episode of breast cancer in relation to the stress variable, adjusting for confounding factors, including age, use of oral contraceptives, any type of hormone replacement, menopause, alcohol intake, smoking, socioeconomic status, and family history of breast cancer.

Studies were excluded if: (a) the articles which not had English version; (b) the articles addressed life style and daily stress; (c) stress was assessed in women with a psychiatric history; or (d) breast cancer recurrence or other diseases of the breast were measured. In addition, review articles and editorials were excluded.

### Strategy for article identification and selection and data collection

The article titles and abstracts were initially evaluated by three reviewers to verify that each primary study addressed the underlying question of the systematic review. The abstracts were grouped into selected versus not selected. The selected articles were retrieved, read in full, and screened for those indexed in more than one source or in another language.

In the next phase, data from the selected studies were assigned to an instrument to verify whether they met the inclusion and exclusion criteria, with discrepancies resolved by discussion and consensus. Studies lacking a consensus for inclusion were analyzed by a fourth reviewer.

Data from the case–control and cohort studies were assigned to a structured form, which included the last name of the first author, the year of publication, country of origin, type of study, adjustment for confounding factors, and odds ratios (ORs) and 95% confidence interval (CI). The data were reviewed by the four reviewers.

### Statistical analysis

Statistical analysis was performed preferentially using Cochrane Review Manager Software (version 5.1). For categorical variables, weighted risk ratios and their 95% CIs were calculated using RevMan 5.1 software [14]. Results were tested for heterogeneity at significance level of *P* < 0.05 as described [15]. A fixed effects model was used if there was no evidence of heterogeneity among studies, whereas a random effects model was used if there was evidence of heterogeneity. The OR and 95% CI for each trial were presented in a Forrest plot. Potential publication bias was assessed by funnel plots, with an asymmetric plot suggesting a possible publication bias. Funnel plot asymmetry was assessed by Egger’s linear regression test using the standardized estimate of the size effect as the dependent variable and the inverse of the standard error as the independent variable. Results were considered statistically significant if *p* < 0.05.

## Results

### Trials and patients

The search strategy identified 307 titles and abstracts. Of these, 284 were excluded after reading the titles and abstracts. Our inclusion and exclusion criteria were applied to the remaining 23 articles describing case–control and cohort studies. A higher intensity of psychological events resulting from severe, major life, stressful, and overall life events were described and classified to calculate the ORs in these articles.

Of the 23 articles, seven, containing sufficient data, were included in our meta-analysis (Table 1). Most of these studies showed satisfactory methodological quality [16]. The cutoff point characterizing these studies as having a high methodological score was the median value of these studies (Table 1). Based on the Downs & Black criteria, the maximum possible total scores were 20 and 18 points for cohort and case–control studies, respectively.

**Table 1:** **Characteristics and downs** &**black scores of studies included in the meta-analysis**

**Authors/Year**	**Country**	**Design**	**Assessment instruments**	**Sample size**	**Age**	**Type of stress**	**Specific events**	**Evaluation moment**	**Disease stage**	**Type of treatment**	**Result RR (95% CI)**	**Score**
Chen 1995 [17]	England	Case–control	4 point scale (great, moderate, some, and little or no)	41/78	20 – 70	Great life events	None	No description	All stages	No description	7.08 (2.31-21.65)	18
Roberts 1996 [18]	America	Case–control	Holmes-Rahe life-event weights	258/614	50 - 79	Stressful life events	Allow for both shorter time of administration and appropriateness (primarily older women)	During the previous 5 years	All stages	Hormone replacement therapy	0.9 (0.78-1.05)	18
Protheroe 1999 [19]	Australia	Case–control	Four point scale, and six point scale for severity difficulties lasting 4 weeks	106/226	40 - 79	Stressful life events	Excluded events that were related to past and present breast problems, or a first degree relative's breast cancer	During the previous 5 years	All stages	Hormone replacement therapy	0.91 (0.47-1.81)	17
										Oral contraceptives		
Kruk 2012 [20]	Poland	Case–control	Holmes-Rahe life-event weights	858/1085	28 - 79	Life events	The association between job stress and breast cancer was determined in separate analysis	During the previous 3 years	All stages	Hormone replacement therapy	5.09 (3.41-8.50)	18
Helgesson 2003 [21]	Sweden	Prospective	1–6 on the stress scale	1462	38 - 60	Stressful events	None	During the previous 5 years	All stages	No description	2.1 (1.2-3.7)	20
Lillberg 2003 [22]	Finland	Prospective	Holmes-Rahe life-event weights	10808	>24	Stressful life events	None	During the previous 5 years	All stages	Oral contraceptives	1.07 (1.00-1.15)	20
Michael 2009 [23]	America	Prospective	Number of life events and amount of upset	84334	50 - 79	Life events	None	During the previous 1 years	All stages	No description	1.12 (1.01-1.25)	19

*RR* relative risk, *CI* confidence interval.

Of the seven studies included in our meta-analysis, four were case–control studies [17-20] and three were cohort studies [21-23]. The four case–control studies were from the United States, Poland, England, and Australia [17-20], with the U.S. study including maximum sized sample. The seven studies included 99,807 women, with age set at higher than 38 years, with one study setting age as more than 50 years.

The remaining 16 identified articles not included in our meta-analysis were examined. Risk factors related to psychiatric, psychological, and social disorders have been described [24]. In addition, the psychological factors and serum biochemical indices defining the association between life events and myeloid-derived suppressor cells were evaluated [25]. Studies have also evaluated the psychosocial approach [26-28], with life events contributing to delays in diagnosis and treatment [28]. Several studies referred to other types of stress (e.g. stresses associated with work, activities of daily life, or lifestyle, as well as post-traumatic stress) [27,29-33]. Indeed, one study found no association between life events and the incidence of breast cancer [34].

### Association between striking life events and the incidence of primary breast cancer

**Figure 1:**
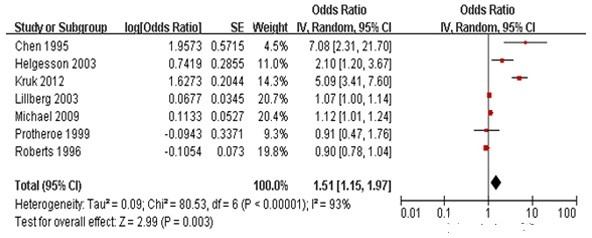
**Meta-analysis of the relative risk, or odds ratio, for the association between striking life events and primary breast cancer incidence.** Solid squares represent risk estimates for the individual studies, with the size of the squares proportional to the sample size and the number of events. Horizontal lines denote 95% confidence intervals (CIs). The diamond shows the confidence interval for the pooled relative risks. Positive values indicate an increased relative risk for primary breast cancer development. Test for overall effect: *Z* = 2.99, *P* < 0.01; chi-square test for heterogeneity = 80.53, degrees of freedom = 6, *P* < 0.001; *I*^2^ = 93%.

ORs for primary breast cancer occurrence related to striking life events are shown in Table 1. In the present study, striking life events was used as a marker of serious psychological events, including stress of life events and great life events. Analysis of ORs values and 95% CIs regarding the association between stressful life events and the risk of breast cancer occurrence varied widely, due to high heterogeneity in the consistency test. We therefore abandoned the fixed effects model, with a random effects model used in the meta-analysis (Figure 1).

The consistency of the seven studies was poor and varied markedly (*p* < 0.00001, Figure 1). Random effects model analysis showed that, in regard to striking life events, the overall OR was 1.51 (95% CI 1.15 - 1.97), indicating that the risk of breast cancer was 1.5-fold higher in populations with than without striking life events (*p* = 0.003). As shown in forest plots, rhombus shapes that represent the studied variable appeared on the right side of invalid line, suggesting the studied variable was a strong pathogenic factor. Interestingly, the rhombus shape suggested that the variable had not been characterized. The OR was far from the midline and differed markedly from other studies. The weight ratio depended on the model used for analysis, with a minimum weight box displayed in the forest plots. The maximal weight box did not represent those reported previously and included the highest number of samples (84,334 cases), although others had difference perspectives (10,808 cases). Although both were prospective cohort studies, subject age was limited from 50 to 79 years, with no specific age limitations.

### Association between severe striking life events and the incidence of primary breast cancer

Of the 7 included studies, three described severe life events. In one study, life events were categorized into those with little or no threat, some threat, moderate threat, and severe threat, depending on subjective human feelings, with the OR of primary breast cancer higher in subjects with severe threat [17]. A second study evaluated severe life events based on scores, finding that OR of primary breast cancer increased from 5.09 to 5.33 as scores increased [20]. In contrast, when severe life events were based on multiple events, the OR for primary cancer decreased from 1.12 for a single event to 0.91 for more than three events [23]. To assess the reasons for these differences, we performed a meta-analysis regarding ORs of severe life events in the included studies because the phrase “severe life events” was close to the connotation of “striking life events” in the present study (Table 2). Because the analysis of Ors showed considerable heterogeneity in consistency tests, the fixed effects model was abandoned and the random effects model was used in our meta-analysis.

**Table 2:** **Characteristics and downs** &**black scores of studies assessing serious striking life events**

**Authors/Year**	**Country**	**Design**	**Valable**	**OR (95% CI)**
Chen 1995 [17]	England	Case–control	Severe life events	11.64 (3.10-43.66)
Protheroe 1999 [19]	Australia	Case–control	Severe life events	0.91 (0.47-1.81)
Kruk 2012 [20]	Poland	Case–control	Major life events	5.33 (4.01-8.21)
Helgesson 2003 [21]	Sweden	Prospective	Stressful events	2.1 (1.2-3.7)
Lillberg 2003 [22]	Finland	Prospective	Major life events	1.35 (1.09-1.67)
Michael2009 [23]	America	Prospective	≥4 life events	0.91 (0.77-1.08)

*RR* relative risk, *CI* confidence interval.

**Figure 2:**
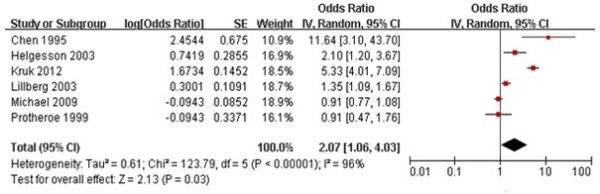
**Meta-analysis of the relative risk, or odds ratio, for the association between severe striking life events and primary breast cancer incidence.** Solid squares represent risk estimates for the individual studies. The size of the squares is proportional to the sample size and the number of events. The horizontal lines denote 95% confidence intervals (CIs). The diamond shows the confidence interval for the pooled relative risks. Positive values indicate an increased relative risk for primary breast cancer incidence. Test for overall effect: *Z* = 2.23, *P* < 0.01; chi-square test for heterogeneity = 123.79, degrees of freedom = 5, *P* < 0.001; *I*^2^ = 96%.

We found that the risk of breast cancer was strongly and significantly associated with more severe striking life events (OR 2.07, 95% CI 1.06 - 4.03, *P* = 0.03), suggesting that individuals with severe striking life events would be at two-fold greater risk of developing breast cancer than individuals without these severe striking life events (Figure 2). In addition, we found that the risk of breast cancer incidence was positively associated with both striking (OR 1.51) and severe striking life events (OR 2.07) in the population.

## Discussion

Primary breast cancer is the most common malignant disease in women. Although many studies have assessed the relationship between the incidence of breast cancer and life events, both epidemiologically and etiologically, the results have been inconsistent [35-37]. Several of these studies reported that life events were significantly associated with breast cancer risk [37,38]. Evidence has emerged showing that these life events may affect the hypothalamic-pituitary-adrenal axis, resulting in endocrine system disorders, increased cortisol concentrations, and reductions in antineoplastic activity [7,8,39]. However, some studies found that stressful life events were not associated with the development of primary breast cancer [40,41]. The first meta-analysis, which included 29 studies, showed a lack of a causal relationship between negative life events and breast cancer incidence [39]. The second meta-analysis, which included 27 studies, assessed several categories of stressful life events, including death of a husband, death of a friend, health problems, financial problems, and change in marital status [41]. Although there was no association between stressful events and breast cancer, there was a slight association between death of a husband and risk of breast cancer. Moreover, it was unclear whether a high degree of depression and anxiety induced by life events, resulting in immune suppression, would promote breast cancer risk, especially when organ transplant recipients who receive immune suppression therapy did not develop multiple malignancies [42-45].

A meta-analysis is a quantitative overview of multiple studies, with evaluation criteria assessing the quality and controlling for selection bias being extremely important. We therefore utilized the Downs & Black method of assessing literature quality to minimize the uneven quality of data collection, criteria used in other meta-analyses and systematic reviews [46-48]. Considering the methodological quality of the reviewed articles, the seven studies included in our meta-analysis were methodologically homogeneous. However, the limitation of populations in some cohort studies to older patients may introduce a selection bias to observed psychological changes after life events. Nevertheless, our meta-analysis selected articles published over 17 years to show that, despite differences in research methods, striking life events remained associated with primary breast cancer incidence.

Psychologically, being in a depressed state and life events are somewhat connected as well as being different. Being depressed is a continuing psychological status, whereas life events are associated with short-term inner feelings and thoughts. An increasing number of retrospective and prospective studies, including a wide range of sample sizes, have shown the importance of the relationship between life events and the occurrence of breast cancer. Among various types of life events, we found that striking life events contributed more to tumor development. Interestingly, severe life events, important life changes, major life events, severe threat events, and great threat events have been used to describe the similar psychological characteristics of striking life events in this study [17-21].

The seven selected studies differed somewhat in their definition of striking life events. One study divided individual feelings into four levels, severe, moderate, some, and little or no, with severe feelings defined as striking life events [17]. A second study defined striking life events as death of a spouse, family member, or friend; sickness of a family member; sickness of the individual (except for cancer); divorce; economic events; self or spouse retirement or unemployment; and moving one’s residence, suggesting that these be considered a standard set of evaluations of striking life events [18]. Since the inclusion of divorce may be open to different interpretations and may result in a lack of significance of the results, we removed this study from our analysis. A third study defined striking life events by their respective scores or as the numbers of events [20]. Although many previous studies have utilized number rather than degree, validation requires larger patient populations.

Our meta-analysis found that women with striking life events were at 1.5-fold higher risk of developing breast cancer than women without these striking life events (combined OR 1.51, 95% CI 1.15 - 1.97). A forest plot showed a diamond shape, with striking life events on the right side of the invalid line, suggesting that striking life events were strongly associated with the incidence of primary breast cancer. However, although our results indicated that striking life events were positively associated with breast cancer occurrence, the OR was not high and the lower limit of the 95% CI was only 1.15.

More importantly, our meta-analysis found that women with a severe degree of striking life events had an OR of 2.07 (95% CI 1.06 - 4.03) of developing breast cancer, suggesting that more severe striking life events contribute to a higher risk of primary breast cancer in women. Our findings suggest that psychological treatment of striking life events may reduce breast cancer occurrence. Discrepancies over the definition of striking life events would change the association between these events and the risk of developing breast cancer.

## Conclusions

Although studies have yielded contradictory results on the association between stress and breast cancer development, our results confirm that high-intensity stress has a borderline association with the development of breast cancer. However, relative to the findings in most of studies that stress can increase the risk of breast cancer, whether those women who had the most aggressive form of breast cancer also had the highest stress levels was unclear, and there is no real way to tell how much stress the women were under before their diagnosis of breast cancer. Obviously, based on that it’s not clear what’s driving the association between stress and breast cancer development, future studies are necessary to elucidate this relationship.

## Authors’ contributions

YL and CW contributed equally to this work. Both designed the study, collected and analyzed data, and wrote the manuscript. YZ, XH, and LP participated in the collecting and analyzing data. GS and KW performed statistical analyses. QS conceived the study, participated in its design, and helped to draft the manuscript. All authors read and approved the final manuscript.

## Competing interests

The authors declare that they have no competing interests.
